# Grape Seed Proanthocyanidin Extract Inhibits Human Esophageal Squamous Cancerous Cell Line ECA109 via the NF-*κ*B Signaling Pathway

**DOI:** 10.1155/2018/3403972

**Published:** 2018-12-17

**Authors:** Fangming Guo, Yunhua Hu, Qiang Niu, Yu Li, Yusong Ding, Rulin Ma, Xianhua Wang, Shugang Li, Jianxin Xie

**Affiliations:** ^1^Department of Public Health, Shihezi University School of Medicine, 832000, China; ^2^Key Laboratory for Xinjiang Endemic and Ethnic Diseases, 832000, China; ^3^Department of Quality Control of Changji Autonomous Prefecture Center for Disease Control and Prevention, 831100, China; ^4^Department of Biochemistry, Shihezi University School of Medicine, 832000, China

## Abstract

Esophageal squamous cell carcinoma is the most common type of squamous cell carcinoma. Grape seed proanthocyanidin extract (GSPE) is considered to exhibit anticancer activity against several different types of cancer. We aimed to determine whether GSPE inhibited esophageal squamous cancerous cells and the possible involvement of NF-*κ*B in this process. The human esophageal squamous cancer cell line ECA109 was treated with GSPE (0–80 *μ*g/mL) and BAY11-7082 (10 *μ*mol/L) for 12, 24, and 48 h. The MTT assay was used to determine cell proliferation; alterations in cell apoptosis were detected by flow cytometry; levels of inflammatory factors interleukin-6 and cyclooxygenase-2 and apoptotic proteins Bax/Bcl-2 were measured by ELISA; qRT-PCR and western blots were used to examine the activation of caspase-3 and NF-*κ*B signaling. GSPE inhibited the proliferation of ECA109 cells and induced cellular apoptosis in a time- and dose-dependent manner. ELISA results showed that GSPE and BAY11-7082 reduced the secretion of inflammatory cytokines interleukin-6 and cyclooxygenase-2. The results of PCR and western blotting indicated that GSPE and BAY11-7082 activated caspase-3 and attenuated the activation of the NF-*κ*B signaling pathway. GSPE induced apoptosis in ECA109 cells and inhibited ECA109 cell proliferation via a reduction in the secretion of inflammatory cytokines. This mechanism may be related to the attenuation of NF-*κ*B activity and the sensitization of caspase-3.

## 1. Introduction

Esophageal carcinoma (EC), one of the most common cancers, is caused by malignant transformation of the esophagus. It is the sixth leading cause of death among malignant cancers, and the most common pathological type is esophageal squamous cell carcinoma (ESCC). The Kazakh area in Xinjiang, China, is a high-risk region for EC. Despite advances in understanding the mechanisms of cancer progression and the development of different therapeutic strategies, EC is still the leading cause of mortality in malignant tumor death among the Kazakh population in Xinjiang, particularly as a result of metastasis [1].

Chronic esophagitis is one of the most important factors for the occurrence of esophageal cancer. Murphy et al. found that non-Barrett's esophagitis increased the risk of ESCC [2]. Zhang et al. reported that local infiltration of inflammatory cells led to the interruption and deletion of the local basement membrane in esophageal squamous cells [3], which promoted cell proliferation and induced EC. Nuclear factor kappa B (NF-*κ*B), a transcription factor that plays an important role in inflammation, is involved in the progress of chronic esophagitis [4]. NF-*κ*B participates in cell proliferation [5], cytoskeletal remodelling [6], cell invasion [7], and apoptosis [8]. Studies have found that NF-*κ*B is a key factor in the development of a variety of malignant carcinomas, such as liver cancer [9], colon cancer [10], and breast cancer [11]. However, a direct connection between NF-*κ*B signaling and EC is less certain.

Proanthocyanidins (PCs), a class of polyphenolic compounds, are widespread in plants, mostly in the epidermis and seeds. Our previous studies determined that PCs reduced oxidative damage and inflammation [12, 13]. Recent research demonstrated the anticarcinogenic activity of PCs [14], with cytotoxic effects reported in various cancerous cell lines (liver [15], colon [16], breast [17], and esophageal [18]) that were largely mediated through apoptosis and showed no adverse biological effects on normal cells. Although it was found that PCs could induce apoptosis in cancer cells, the role of NF-*κ*B in the reversal of EC, as well as the mechanism, remains unclear. Therefore, we conducted this study to determine whether GSPE induced apoptosis in esophageal cancer cells and examined any possible involvement of NF-*κ*B in the process.

## 2. Materials and Methods

### 2.1. Reagents

GSPE (≥95.0%) was obtained from JF-Natural Company (Tianjin, China). BAY 11-7082 and antibodies against IKK, caspase-3, and NF-*κ*B (p65) were supplied by Abcam (Cambridge, England), and antibodies against I*κ*B, phospho-I*κ*B (p-I*κ*B), and NF-*κ*B (p100/p50) were procured from Cell Signaling Technology Inc. (Danvers, MA). Antibodies against GAPDH were purchased from Goodhere Biotechnology (Hangzhou, China). Dulbecco's modified Eagle's medium (DMEM), penicillin, streptomycin, fetal bovine serum (FBS), and trypsin/EDTA were purchased from HyClone (Logan, Utah). 3-(4,5-Dimethylthiazol-2-yl)-3, 5-diphenyltetrazolium bromide (MTT) was obtained from Jiancheng Biotechnology Co. (Nanjing, China). The annexin V-FITC/PI apoptosis kit was procured from Multisciences (Hangzhou, China). ELISA kits for IL-6 and COX-2 were purchased from Elabscience (Wuhan, China).

### 2.2. Cell Culture

Human esophageal squamous ECA109 cells were kindly provided by the Department of Pathology, Key Laboratory for Xinjiang Endemic and Ethnic Diseases, Shihezi University School of Medicine (Xinjiang, China). All cells were cultured in monolayers with 90% DMEM supplemented with 10% FBS and 1% penicillin/streptomycin at 37°C in a humidified atmosphere of 5% CO_2_. The medium was changed every second day.

### 2.3. Cell Viability Assay

The MTT assay was used to measure the viability of ECA109 cells. The cells were plated into 96-well plates at a density of 2000 cells/well in 200 *μ*L DMEM. After incubation at 37°C overnight, GSPE (0–400 *μ*g/mL) was added to the cells for 12, 24, and 48 h. Each treatment and time point were assayed in triplicate. After the stipulated treatment time with GSPE, MTT was added to the cells for 4 h. Subsequently, the supernatant was discarded and the formazan precipitates were dissolved in 150 *μ*L dimethyl sulfoxide (DMSO). An automatic microplate spectrophotometer was used to measure the optical density (OD) for each well. The detected wavelength was 490 nm, and the reference wavelength was 620 nm.

### 2.4. Annexin V-FITC/PI Staining

Apoptosis was determined in ECA109 cells by using an annexin V-FITC/PI apoptosis kit. After treatment with GSPE (0, 25, 50, and 80 *μ*g/mL) for 24 h, the cells were collected and washed twice with cold PBS. Subsequently, 1 × 10^6^ cells were suspended in binding buffer, stained with annexin V-FITC and PI, and analyzed by using flow cytometry.

### 2.5. Cell Migration Assay

The effect of GSPE on ECA109 migration was analyzed by using a cell scratch test. Cells were plated into 6-well plates at a density of 5 × 10^6^ cells/well in 2 mL DMEM supplemented with 10% FBS. The cells were allowed to adhere, scratched by pipette tips, and treated with GSPE (0, 25, 50, and 80 *μ*g/mL) for 24 h. Each treatment was assayed in triplicate. After incubation at 37°C overnight, the cells were observed by using an inverted microscope.

### 2.6. Cell Invasion Assay

A Transwell cell invasion assay was performed. Briefly, the upper chamber of Millicell cell culture inserts was coated with 50 *μ*L Matrigel diluted 1 : 8 with PBS. Subsequently, 4 × 10^5^ ECA109 cells in 0.4 mL serum-free DMEM, with or without GSPE, were added to the upper chamber. The lower chamber was filled with 0.6 mL DMEM supplemented with 20% FBS as a chemoattractant to induce invasion. After incubation at 37°C for 24 h, the culture inserts were removed and the noninvasive cells on the upper surface of the culture inserts were removed by using a cotton swab. The cells that invaded through the Matrigel were fixed with methanol for 30 min and stained with 0.1% crystal violet for 10 min at 20°C. Images were captured by using light microscopy.

### 2.7. ELISA

Briefly, the cells were cultured with GSPE (0, 25, 50, and 80 *μ*g/mL) and GSPE (0, 25, 50, and 80 *μ*g/mL) + BAY11-7082 (10 *μ*mol/L) for 12, 24, and 48 h. Supernatants from experimental cultures were collected and stored at −80°C until use. The levels of IL-6 and COX-2 in the supernatants were determined by using cytokine detection ELISA kits in accordance with the manufacturer's instructions; detection at 450 nm was conducted by using a microplate reader. The concentration of Bax and Bcl-2 in the cell culture supernatant was determined by using a Bax and Bcl-2 detection ELISA kit.

RT-PCR was performed to evaluate the mRNA expression of caspase-3, IKK, NF-*κ*B (p50), and NF-*κ*B (p65) after treatment with GSPE (0, 25, 50, and 80 *μ*g/mL) and GSPE (0, 25, 50, and 80 *μ*g/mL) + BAY11-7082 (10 *μ*mol/L) for 24 h, as previously described [19]. The designed primers are shown in Table 1.

### 2.8. Western Blot Analysis

ECA109 cells were treated with GSPE (0, 25, 50, and 80 *μ*g/mL) and GSPE (0, 25, 50, and 80 *μ*g/mL) + BAY11-7082 (10 *μ*mol/L) for 24 h. After treatment, the cells were collected and washed three times with PBS. The harvested cells were lysed on ice for 30 min in 100 mL of lysis buffer. The total protein was collected and quantified by using the Bradford assay. The separated proteins were transferred onto nitrocellulose membranes, which were first incubated with antibodies against caspase-3, IKK, phospho-I*κ*B, I*κ*B, NF-*κ*B (p50), NF-*κ*B (p65), and GAPDH, and then incubated with secondary anti-mouse or anti-rabbit antibodies. All western blotting studies were repeated three times.

### 2.9. Statistical Analysis

All values are expressed as the mean ± standard deviation (SD), and analyses were computed by using SPSS 20.0. Western blotting analysis was calculated by using Image-Pro Plus software. The comparison of the mean among multiple groups was performed with analysis of variance. Pairwise comparison among groups was performed with the least significant difference (LSD) tests. For all preplanned or a priori contrasts stipulated in the main hypotheses, a significance level of 0.05 or 0.01 was considered to indicate statistical significance.

## 3. Results

### 3.1. GSPE Inhibited the Survival of ECA109 Cells

**Figure 1:**
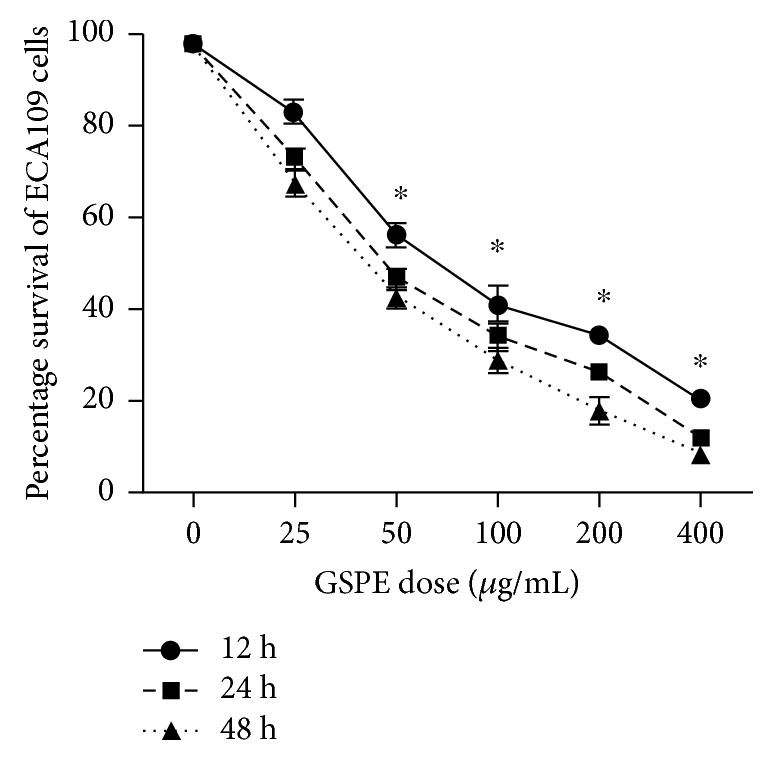
The effect of GSPE on ECA109 survival. The cytotoxicity of GSPE (0–400 *μ*g/mL and 12–72 h) was detected by MTT assay. Each column represented mean ± SD of three groups of independent samples. ∗ means *P* < 0.05 compared with GSPE 0 *μ*g/mL.

GSPE exerted an obvious inhibitory effect on ECA109 cell survival, as shown in Figure 1. A higher GSPE dose resulted in a stronger inhibitory effect on ECA109 cells; similarly, a higher application time for a specific GSPE dose significantly decreased the survival rate of ECA109 cells (*P* < 0.05). GSPE had a significant time- and dose-dependent inhibitory effect on ECA109 cells. Through the calculation of IC_50_ after the application of GSPE for different times, we selected the treatment doses of GSPE as 25, 50, and 80 *μ*g/mL (Table 2). In addition, our results showed that the survival rate of ECA109 was decreased by the intervention of GSPE for 24 h and 48 h, but the difference was not statistically significant.

### 3.2. GSPE Induced Apoptosis in ECA109 Cells

**Figure 2:**
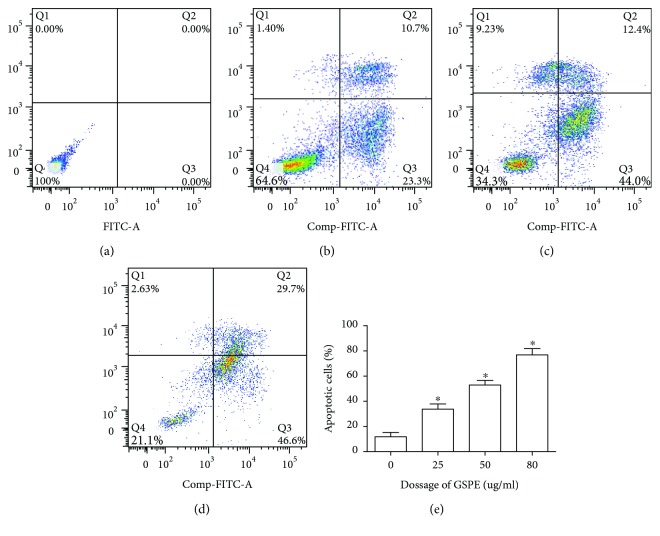
GSPE induced apoptosis of esophageal cancer cell ECA109 apoptosis. ECA 109 cells were treated with GSPE (0–80 *μ*g/mL) for 24 h. Double staining with annexin V-FITC and PI was used to determine apoptosis. Values were mean ± SD of three independent samples. (a) GSPE 0 *μ*g/mL; (b) GSPE 25 *μ*g/mL; (c) GSPE 50 *μ*g/mL; (d) GSPE 80 *μ*g/mL; (e) GSPE 0–80 *μ*g/mL. ^∗^*P* < 0.01 compared with the GSPE 0 *μ*g/mL group.

We used flow cytometry to determine whether GSPE affected the apoptosis of ECA109 cells. Between GSPE concentrations of 25, 50, and 80 *μ*g/mL, the percentage of apoptotic ECA109 cells increased from 34.0% to 76.3% and the differences between each group were statistically significant (*P* < 0.05). In this experiment, we used FITC and PI double staining. In the histogram, the first quadrant represents the cells in late apoptosis and the second quadrant represents the cells in early apoptosis. We found that the application of GSPE (25–80 *μ*g/mL) for 24 h increased the percentage of ECA109 cells in early apoptosis and in late apoptosis (*P* < 0.05); furthermore, a dose-dependent relationship was found (Figure 2).

### 3.3. GSPE Inhibited ECA109 Cell Migration

**Figure 3:**
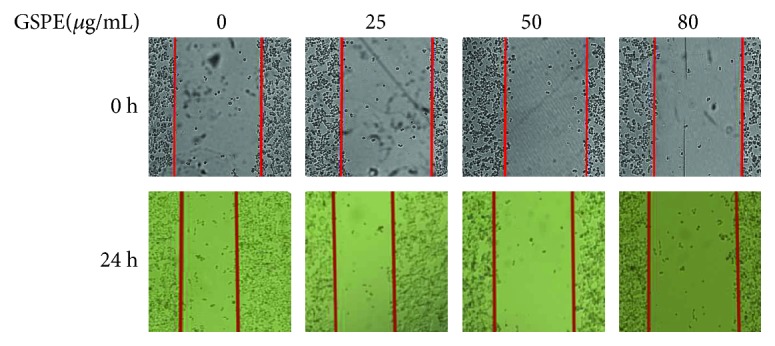
The effect of GSPE on the migration of ECA109. The effect of GSPE (0–80 *μ*g/mL) on ECA109 migration was analyzed by using a cell scratch test. Each treatment was assayed in triplicate. After incubation at 37°C for 24 h, the cells were observed by an inverted microscope (magnification, ×100).

Based on the results of the MTT and flow cytometry assays, we observed the change in cell migration capacity after GSPE treatment for 24 h. For 25 *μ*g/mL GSPE, the change in cell migration distance was not obvious compared with that in the control, but at 50 and 80 *μ*g/mL, the distance was significantly shortened (Figure 3).

### 3.4. GSPE + BAY11-7082 Inhibited the Invasion of ECA109 Cells

**Figure 4:**
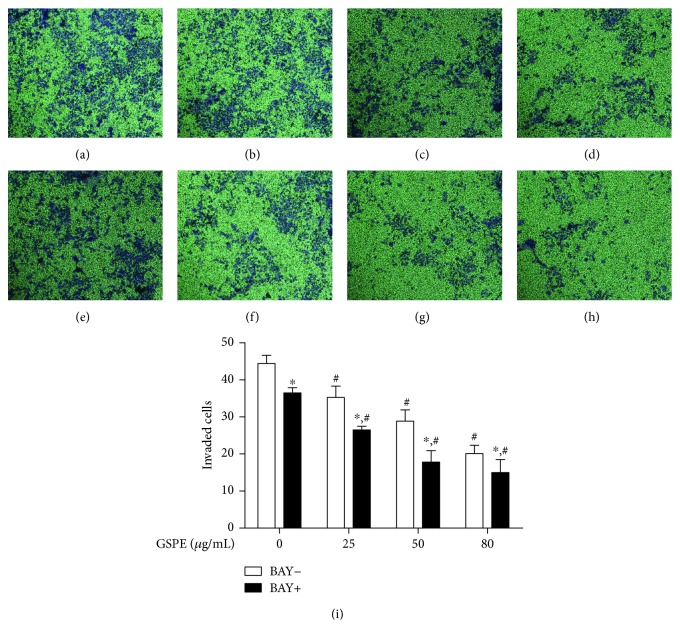
The effect of GSPE and BAY11-7082 on the invasion ability of ECA109 cells. The effect of GSPE on ECA109 invasion was analyzed by using Transwell assay. Each treatment was assayed in triplicate. After incubation at 37°C for 24 h, the cells were observed by an inverted microscope (magnification, ×100). (a–d) The inhibition of invasion ability in cells induced by GSPE (0–80 *μ*g/mL). (e–h) The inhibition of invasion ability in cells induced by GSPE (0–80 *μ*g/mL) + BAY11-7082. (i) The number of invaded cells, which was evaluated by ImageJ 2x. Each column represents mean ± SD of three groups of independent samples. ^∗^*P* < 0.05 compared with the BAY11-7082 group; ^#^*P* < 0.05 compared with the GSPE 0 group.

Compared with the control group, the application of GSPE (25, 50, and 80 *μ*g/mL) reduced the number of cells that passed through the well (Figures 4(a)–4(d)). It was suggested that the inhibitory effect on ECA109 cells was elevated with the increasing concentration of GSPE, while the invasive abilities of ECA109 cells were decreased.

After the simultaneous application of GSPE (0, 25, 50, and 80 *μ*g/mL) and 10 *μ*mol/L BAY11-7082 to the Transwell chambers, the cultured cells were observed after 24 h (Figures 4(e)–4(h)). Compared with the control group, all concentrations of GSPE + BAY11-7082 inhibited cell movement through the Transwell chambers (Figure 4(i)).

### 3.5. GSPE and BAY11-7082 Inhibited Inflammatory Cytokine Levels in ECA109 Cells

**Figure 5:**
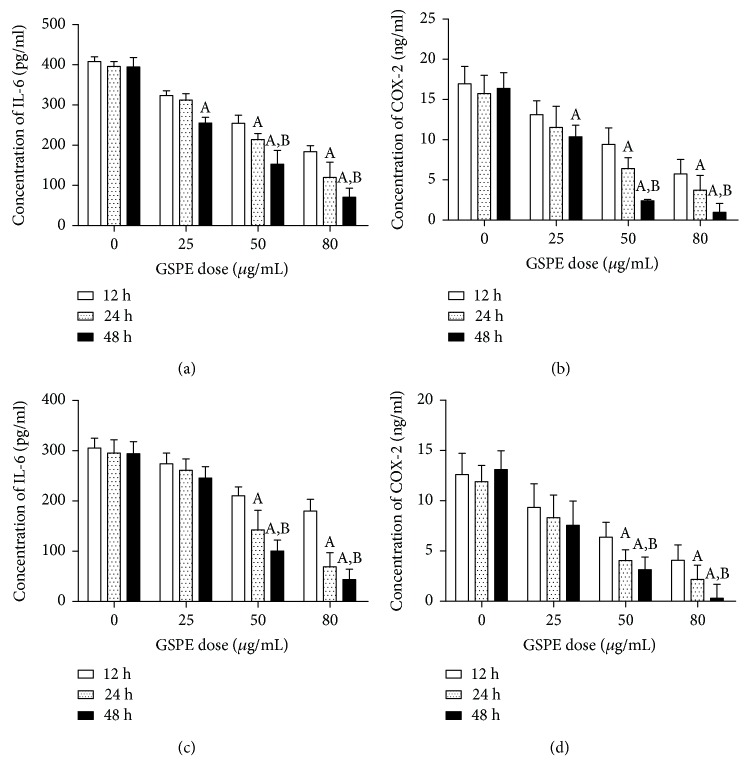
GSPE and BAY11-7082 inhibited the expression of inflammatory cytokines IL-6 and COX-2. (a, b) The inhibition of IL-6 and COX-2 in cells induced by GSPE (0–80 *μ*g/mL). (c, d) The inhibition of IL-6 and COX-2 in cells induced by GSPE (0–80 *μ*g/mL) + BAY11-7082. Each column represents mean ± SD of three groups of independent samples. ^A^*P* < 0.05 compared with the 12 h group; ^B^*P* < 0.05 compared with the 24 h group.

In the absence of GSPE, a high level of secretion of IL-6 and COX-2 was observed in ECA109 cells. In the presence of GSPE, the secretion of IL-6 and COX-2 in the cells was inhibited; an increase in GSPE dose led to more obvious inhibition (*P* < 0.05) (Figures 5(a) and 5(b)). In addition, we observed the effect of the same GSPE dose applied for different times on the secretion of IL-6 and COX-2 and found that stronger inhibition occurred when the same GSPE dose was applied for longer times (*P* < 0.05). The measurement of the concentration of IL-6 and COX-2 in ECA109 cells after treatment with GSPE + BAY11-7082 showed that GSPE + BAY11-7082 could inhibit the secretion of inflammatory cytokines in ECA109 cells; furthermore, the inhibitory effect of GSPE + BAY11-7082 was stronger than that caused by GSPE treatment alone (Figures 5(c) and 5(d)).

### 3.6. GSPE and BAY11-7082 Promoted Bax and Inhibited the Activity of Bcl-2

**Figure 6:**
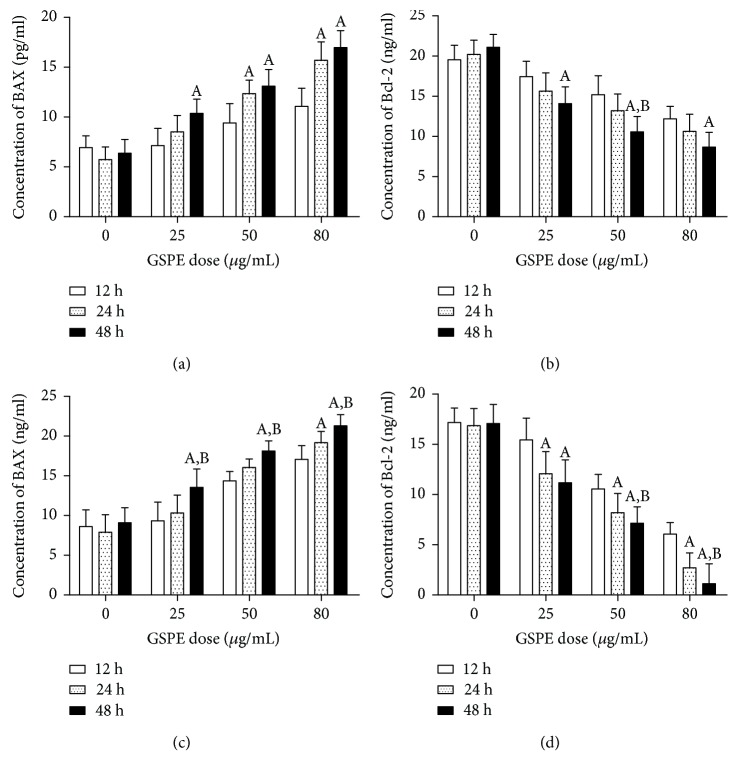
GSPE and BAY11-7082 inhibited Bax/Bcl-2 expression in ECA109 cells. (a, b) GSPE inhibited Bax and Bcl-2 in cells. (c, d) GSPE + Bay11-7082 inhibited Bax and Bcl-2 in cells. Each column represents the mean ± SD of three groups of independent samples. ^A^*P* < 0.05 compared with the 12 h group; ^B^*P* < 0.05 compared with the 24 h group.

We investigated the effects of different times and different doses of GSPE compared with the control group. The protein levels of Bax increased and the protein levels of Bcl-2 decreased; a time- and dose-dependent relationship was observed (Figures 6(a) and 6(b)). The same changes were found when different concentrations of GSPE and 10 *μ*mol/mL BAY11-7082 were simultaneously applied (Figures 6(c) and 6(d)).

### 3.7. GSPE and BAY11-7082 Activated Caspase-3

**Figure 7:**
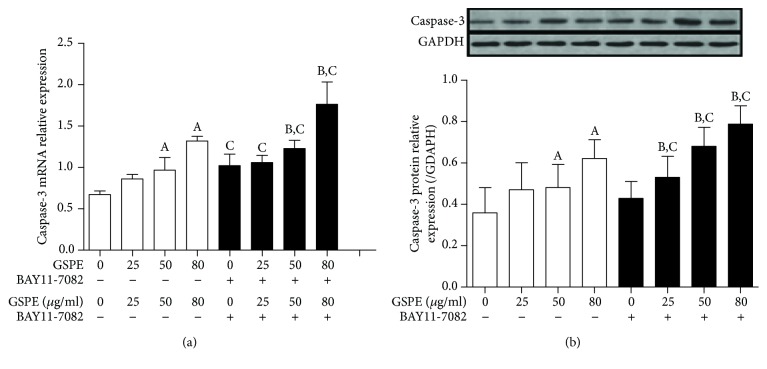
The effects of GSPE and BAY11-7082 on the expression of caspase-3 mRNA and protein in ECA109 cells. (a) GSPE (0–80 *μ*g/mL) and BAY11-7082 (10 *μ*mol/L) inhibited the expression of caspase-3 mRNA; (b) caspase-3 protein was inhibited by GSPE (0–80 *μ*g/mL) and BAY11-7082 (10 *μ*mol/L). Each column represents the mean ± SD of three groups of independent samples. ^A^*P* < 0.05 compared with the 12 h group; ^B^*P* < 0.05 compared with the 24 h group; ^C^*P* < 0.05 compared with the BAY11-7082 group.

We examined the effects of GSPE and BAY11-7082 on the mRNA and protein expression of caspase-3 by using PCR and western blotting, respectively. In untreated ECA109 cells, the mRNA and protein expression of caspase-3 occurred at a relatively low level. With an increased dose of GSPE and the addition of Bay11-7082, the expression level of caspase-3 mRNA and protein increased (Figures 7(a) and 7(b)). This suggested that GSPE and BAY11-7082 promoted the apoptosis of ECA109 cells through the activation of caspase-3.

### 3.8. GSPE and BAY11-7082 Inhibited the NF-*κ*B Pathway

In view of the important role of NF-*κ*B in the regulation of cytokines and the induction of apoptosis, we studied the effect of GSPE and BAY11-7082 on the transcription factors. We used western blotting to detect the protein expression levels of various classical factors, including IKK, I*κ*B, p-I*κ*B, p50, and p65, in the NF-*κ*B pathway.

**Figure 8:**
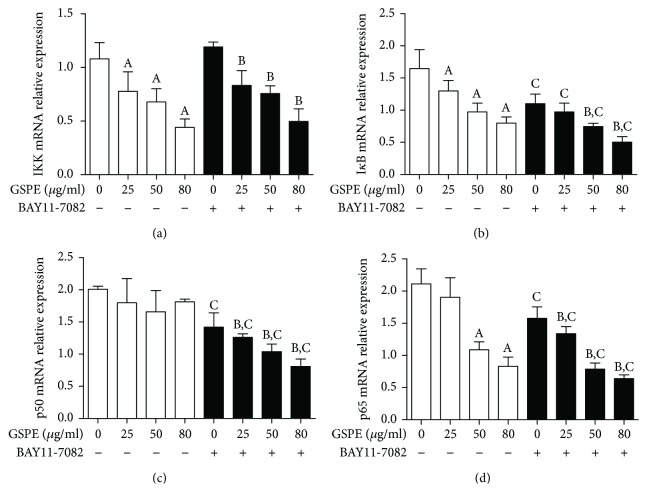
The effects of GSPE and BAY11-7082 on the expression of IKK, I*κ*B, p50, and p65 mRNA in ECA109 cells. (a) The inhibition of IKK mRNA expression by GSPE (0–80 *μ*g/mL) and BAY11-7082 (10 *μ*mol/L). (b) The inhibition of I*κ*B mRNA expression by GSPE (0–80 *μ*g/mL) and BAY11-7082 (10 *μ*mol/L). (c) The inhibition of p50 mRNA expression by GSPE (0–80 *μ*g/mL) and BAY11-7082 (10 *μ*mol/L). (d) The inhibition of the p65 mRNA by GSPE (0–80 *μ*g/mL) and BAY11-7082 (10 *μ*mol/L). Each column represents the mean ± SD of three groups of independent samples. ^A^*P* < 0.05 compared with the GSPE 0, BAY11-7082; ^B^*P* < 0.05 compared with the GSPE 0, BAY11-7082+ group; ^C^*P* < 0.05 compared with the BAY11-7082 group.

**Figure 9:**
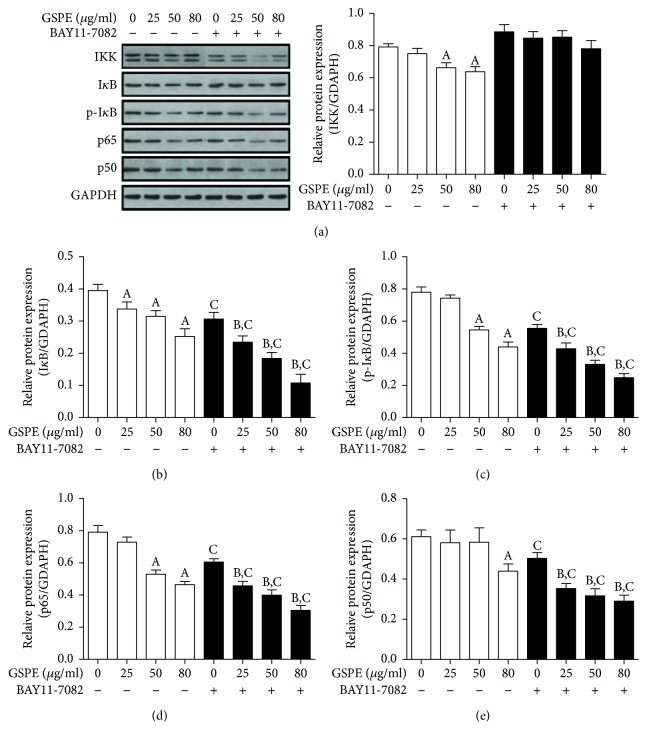
The effects of GSPE and BAY11-7082 on the expression of IKK, I*κ*B, p50, and p65 protein ECA109 cells. (a) The inhibition of the IKK protein expression by GSPE (0–80 *μ*g/mL) and BAY11-7082 (10 *μ*mol/L). (b) The inhibition of I*κ*B protein expression by GSPE (0–80 *μ*g/mL) and BAY11-7082 (10 *μ*mol/L). (c) The inhibition of p-I*κ*B protein expression by GSPE (0–80 *μ*g/mL) and BAY11-7082 (10 *μ*mol/L). (d) The inhibition of p65 protein expression by GSPE (0–80 *μ*g/mL) and BAY11-7082 (10 *μ*mol/L). (e) The inhibition of p50 protein expression by GSPE (0–80 *μ*g/mL) and BAY11-7082 (10 *μ*mol/L). The mean ± SD of three groups of independent samples are shown in each column. ^A^*P* < 0.05 compared with the GSPE 0, BAY11-7082–; ^B^*P* < 0.05 compared with the GSPE 0, BAY11-7082+ group; ^C^*P* < 0.05 compared with the BAY11-7082 group.

In the absence of any treatment interventions, we observed that the protein expression of various transcription factors in ECA109 cells was at a high level, which indicated the activation of NF-*κ*B signaling pathway in esophageal cancer cells. However, the mRNA and protein expression levels of IKK, I*κ*B, p-I*κ*B, and p65 were decreased after treatment with 25, 50, and 80 *μ*g/mL for 24 h, whereas the mRNA and protein expression levels of p50 and p65 were increased (Figures 8 and 9). Similar results were found when GSPE and BAY11-7082 were simultaneously applied to ECA109 cells. However, we found that the treatment of BAY11-7082 alone did not result in a decrease in IKK mRNA levels (Figure 8(a)).

## 4. Discussion

Esophageal cancer is one of the most common malignant tumors in China. The incidence of EC in the Kazakh population of Xinjiang, China, is increasing. A clinical operation is the most common treatment for this disease, but the recurrence rate is high owing to the high metastasis rate of EC [20]. Therefore, it is essential to explore effective natural plant drugs and molecular therapeutic targets that induce apoptosis and inhibit the mechanisms of cell migration and metastasis. In this study, the survival rate of ECA109 cells was determined in the presence of different concentrations of GSPE. GSPE was found to inhibit the proliferation of ECA109; as the dose increased, a stronger effect was observed on the migration and invasion of esophageal cancer cells. These inhibitory effects were accompanied by the decreased secretion of inflammatory factors such as IL-6, CRP, COX-2, and prostaglandin E2 (PGE2); Bax activation; Bcl-2 inhibition; the activation of caspase-3; and inhibition of the NF-*κ*B pathway.

IL-6, similar to many core inflammatory factors, is increased by a large amount in the inflammatory microenvironment of cancer cells; this occurs through the induction of CRP, which activates the NF-*κ*B pathway to reduce the activity of caspase-3 and inhibit the apoptosis of cancer cells [21]. In contrast, the activation of extracellular matrix degradation enzymes can promote the migration and invasion of cancer cells [17]. In this study, GSPE decreased the secretion of inflammatory cytokines (IL-6 and COX-2) in cells, causing the inhibition of the growth, proliferation, migration, and invasion of ECA109 cells. A high level of IL-6 and COX-2 is closely related to the growth [19], migration [22], and invasion [23] of cancer cells. COX-2 is considered to be the rate-limiting enzyme for the conversion of arachidonic acid into prostaglandin E2 (PGE2), which is often expressed in tissue damage or inflammatory response. In vitro experiments indicated that COX-2 was highly expressed in esophageal cancer [24], liver cancer [25], and endometrial cancer [26] and that a higher COX-2 level resulted in higher cell proliferation. In the correlation analysis of COX-2 and Bax in cancer cells of patients with renal cancer, a negative correlation was found. It is believed that COX-2 promotes the proliferation of cancer cells through the inhibition of Bax activity [27]. Therefore, we hypothesized that GSPE induced apoptosis in ECA109 cells through the activation of caspase-3 and the inhibition of Bax via the inhibition of the expression of inflammatory cytokines. This was confirmed by the measurement of the mRNA and protein levels of caspase-3.

The NF-*κ*B signaling pathway is involved in the occurrence and development of a variety of malignant tumors [28]. NF-*κ*B exerts antiapoptotic activity mainly by influencing the expression of various inflammatory factors, such as IL-6 and COX-2, and effectors, such as Bax/Bcl-2 and caspase-3. The study found that GSPE prominently inhibited the protein expression of p-I*κ*B in ECA109 cells and prominently promoted I*κ*B mRNA and protein expression, which implied that the GSPE inhibition of NF-*κ*B may be predominantly realized through the inhibition of I*κ*B phosphorylation. Terra et al. used procyanidins B1 and C1 to interfere with LPS-induced macrophages and found that the proanthocyanidins inhibited the activation of the NF-*κ*B pathway by inhibiting the phosphorylation of I*κ*B [29]. However, Zhao et al. found that GSPE inhibited I*κ*B in human ovarian cancer A2780 cells, which inhibited the NF-*κ*B pathway and subsequently promoted apoptosis [15]. Based on the effects of GSPE, we also investigated the treatment of the NF-*κ*B-specific inhibitor BAY11-7082 and found that GSPE + BAY11-7082 was a more effective inhibitor of the phosphorylation level of I*κ*B compared with GSPE alone. This suggested that the inhibition of NF-*κ*B by GSPE was achieved by the inhibition of I*κ*B phosphorylation; a similar effect occurred with BAY11-7082, showing that GSPE and BAY11-7082 may have a synergistic inhibitory effect on the NF-*κ*B in ECA109 cells.

In addition, we found that GSPE inhibited the expression of NF-*κ*B p50/p65 mRNA and protein in cells. NF-*κ*B p50/p65, the most common heterogeneous dimer in the NF-*κ*B signaling pathway, is also an important protein for the function of NF-*κ*B. In resting cells, NF-*κ*B p50/p65 and I*κ*B form complexes, which exist in the cytoplasm in an inactive form. When the cell is stimulated by an extracellular signal, the I*κ*B kinase complex (IKK) activates the phosphorylation of I*κ*B, and the NF-*κ*B is exposed to the nuclear localization site. The dissociated NF-*κ*B is rapidly shifted to the nucleus, binding to a specific *κ*B sequence and inducing the transcription of related genes. The GSPE inhibition of NF-*κ*B p50/p65 resulted from the inhibition of I*κ*B phosphorylation by GSPE, which was consistent with the research of Mackenzie et al. [30]. Some studies have suggested that the ability of procyanidins to inhibit NF-*κ*B p50/p65 expression inhibition may result from the appearance of the procyanidin dimers that may mimic the arginine residues of the NF-*κ*B p50/p65 sequence, with respect to hydrogen bonding, to inhibit the expression of p50/p65 [31]. However, our study did not indicate whether the chemical structure of GSPE was related to the expression of NF-*κ*B p50/p65.

In addition, we found that the mRNA and protein expressions of IKK were both inhibited by GSPE. However, there was no significant difference between the GSPE group and the GSPE + BAY11-7082 group. BAY11-7082, a specific inhibitor of NF-*κ*B, inhibits the phosphorylation of I*κ*B. Therefore, our findings also suggest that GSPE may directly affect IKK, inhibit the activation of IKK, and inhibit the phosphorylation of I*κ*B; together, this inhibits the NF-*κ*B pathway.

**Figure 10:**
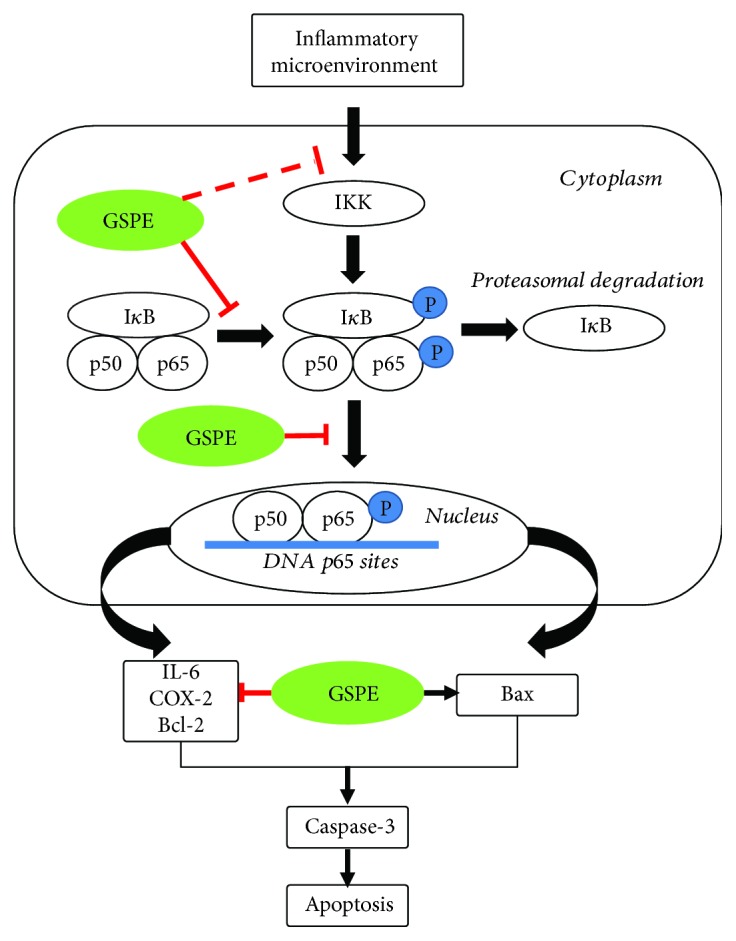
GSPE induced apoptosis in ECA109 cells via NF-*κ*B signaling.

In general, the NF-*κ*B signaling pathway plays an important role in the inhibition of the growth of ECA109 cells by GSPE. GSPE promotes the activation of the apoptotic proteins Bax and caspase-3 through the inhibition of NF-*κ*B pathway activation and the inhibition of the expression of antiapoptotic proteins and inflammatory cytokines, thereby inhibiting the proliferation, migration, and invasion of the ECA109 cell line by the induction of apoptosis (Figure 10).

## 5. Conclusions

Our study has illustrated a possible molecular mechanism for the action of GSPE against cancer; however, the occurrence and development of cancer and the migration and invasion of cancer cells are complex and involve multiple factors. Therefore, the specific mechanism requires extensive research to explore the anticancer effect of procyanidins and provide a basis for their effective use. The results and discussion may be presented separately, or in one combined section, and may optionally be divided into headed subsections.

## Figures and Tables

**Table 1:** Primer of caspase-3 and NF-*κ*B-related factor.

Name	Primer	Sequence
Homo-GAPDH	Forward	5′-TCAAGAAGGTGGTGAAGCAGG-3′
	Reverse	5′-TCAAAGGTGGAGGAGTGGGT-3′
Homo-IKK	Forward	5′-TGTACCAGCATCGGGAACTT-3′
	Reverse	5′-TCAGGAACATCACAGGCCTT-3′
Homo-I*κ*B	Forward	5′-ACTCCCGACACCAACCATAC-3′
	Reverse	5′-CTCCGGTTTGTCAAGGTCAG-3′
Homo-NF-*κ*BP65	Forward	5′-ACCGGATTGAGGAGAAACGT-3′
	Reverse	5′-ACGTAAAGGGATAGGGCTGG-3′
Homo-NF-*κ*BP50	Forward	5′-TCGTTTCCGTTATGTATGTGAAGG-3′
	Reverse	5′-TGTCCTTGGGTCCAGCAGTT-3′
Homo-caspase-3	Forward	5′-ACTGGACTGTGGCATTGAGA-3′
	Reverse	5′-GCACAAAGCGACTGGATGAA-3′

**Table 2:** IC_50_ of GSPE over different treatment times.

GSPE	Duration (h)		
	12	24	48
IC_50_ (*μ*g/mL)	66.442 ± 13.54	51.713 ± 12.69	37.158 ± 13.07

## Data Availability

The datasets used or analyzed during the current study are available from the corresponding author on reasonable request.
